# Shelf Temperature and Depressurisation Strategy Affect Bioactive Retention and Functionality of Freeze-Dried Pumpkin Powder

**DOI:** 10.3390/antiox15080927

**Published:** 2026-07-26

**Authors:** Daniela Sumczynski, Libor Červenka, Helena Velichová, Ludmila Hrabalíková, Jiří Mlček

**Affiliations:** 1 Department of Food Analysis and Chemistry, Faculty of Technology, Tomas Bata University in Zlín, Vavrečkova 5669, 760 01 Zlín, Czech Republic; sumczynski@utb.cz (D.S.); velichova@utb.cz (H.V.); 2 Department of Analytical Chemistry, Faculty of Chemical Technology, University of Pardubice, 532 10 Pardubice, Czech Republic; 3 Department of Clinical Subspecialities, Faculty of Health Studies, University of Pardubice, Průmyslová 395, 532 10 Pardubice, Czech Republic; ludmila.hrabalikova@upce.cz

**Keywords:** HPLC analysis, phenolic, carotenoid, powder flowability, ANOVA, principal component analysis

## Abstract

Background: Pumpkin is a source of phenolics and carotenoids, but their retention during freeze-drying may depend on the drying programme. Methods: Pumpkin flesh was freeze-dried using two schedules: a stepwise schedule (SWS; gradual pressure decrease and shelf heating) or a rapid schedule (RS; deep vacuum and direct shelf heating). Final shelf temperatures of 20, 30 and 40 °C were tested. The powders were analysed for phenolic and carotenoid profiles, residual moisture, colour and basic powder functionality. Results: RS produced lower residual moisture (4.7–5.4%) than SWS (6.2–8.8%). Flowability remained poor but improved at higher shelf temperatures. Water-holding capacity increased, while swelling decreased. Higher levels of protocatechuic acid (121–130 μg/g dry weight), (-)-epicatechin (36–39 μg/g dry weight), and ethyl protocatechuate (7.7–8.8 μg/g dry weight) were determined in samples dried at the lowest shelf temperature, regardless of the drying schedule. SWS30 and SWS40 were associated with caffeic acid, syringic acid and trans-2-hydroxycinnamic acid, and all SWS samples had higher quercetin content. RS gave higher carotenoid levels at 20 °C, while responses at 30 and 40 °C depended on the compound. Conclusions: Final shelf temperature and drying schedule affected antioxidant retention and functional properties of pumpkin powder.

## 1. Introduction

Pumpkin (*Cucurbita* spp.) was selected as the raw material because its flesh is recognised as a source of antioxidant-related phytochemicals, notably carotenoids (with β-carotene, α-carotene, lutein and zeaxanthin frequently reported as major constituents) and as a pool of phenolic compounds that together underpin both nutritional value and colour attributes [1,2]. Pumpkin flesh can be processed into powder to improve its practical use. In this form, the material can be easily stored and transported, and it may have a longer shelf life than fresh flesh. Pumpkin powder can also be added to various food products such as cookies [3], beverages [4] and chips [5]. Freeze-drying was chosen as the dehydration method because water is removed mainly by sublimation at low temperature. Compared with more intensive thermal drying methods, it is generally suitable for producing high-quality dried products with better preservation of structure and sensitive compounds [6,7,8,9]. Nevertheless, the final quality still depends on the design of the freeze-drying cycle, including the time, temperature and pressure profiles [10,11]. Because pumpkin contains both carotenoids and phenolic compounds, its powder is a relevant material for evaluating the effect of freeze-drying conditions on those antioxidants. Carotenoids are sensitive to oxidation, isomerization and heat [4,12,13] and phenolics may also change through oxidative reactions [14,15]. Therefore, the drying programme can influence both the retention and the detailed profile of these compounds, as well as the functional behaviour of the resulting powder.

During freeze-drying, the setting of shelf temperature and chamber pressure are important because they control the amount of heat supplied to the product and the rate of sublimation. If these conditions are not well adjusted, product temperature and drying rate may change, which can influence drying time, structure, colour and the retention of sensitive compounds [11,16,17,18,19,20,21]. For example, a working pressure of 30 Pa and 50 °C for a heating plate temperature were reported as optimal for strawberry drying, keeping the comparable quality while reducing drying time relative to industrial-scale conditions [21]. Recent studies with food materials also indicate that increasing shelf temperature can shorten the process [11,16,17], but the effect on structure and physical powder properties depends on the material and drying conditions. Bogusz et al. found that increasing shelf temperature from 10 to 40 °C during freeze-drying decreased the hygroscopicity and porosity of foam-mat kiwiberry, while increasing its dry matter content and solubility [16]. Shelf temperature during freeze-drying was also determined as the key variable affecting specific surface area [22], mechanical properties [19] or reconstitution behaviour [20,23]. Regarding the retention of bioactive compounds, the combined effect of both shelf temperature and drying time should be considered. Usually, the higher the shelf temperature, the shorter the drying time applied for moisture reduction. Vitamin C is the most frequently studied heat-sensitive compound showing both stability after freeze-drying at different shelf temperatures [17] and better preservation at higher shelf temperatures [19,20,24]. Studies on the effects of freeze-drying conditions (shelf temperature, chamber pressure) on other bioactive compounds are mostly limited to total phenolic content, total anthocyanin content [18,20,24,25], and β-carotene [20,26]. To the best of our knowledge, detailed information on how freeze-drying conditions affect individual phenolic compounds and carotenoids is still limited.

Many food freeze-drying studies are based on constant shelf-temperature and pressure settings. A stepwise increase in shelf temperature can heat the product more gradually at the beginning of drying and may help to limit shrinkage, as observed in freeze-dried strawberries [18]. However, another study found that shrinkage in strawberries was associated with operating pressure rather than shelf temperature [21]. Jakubczyk and Nowak concluded that the choice of shelf temperature and chamber pressure should be based not only on reducing drying time, but also on the expected properties of dried products [11].

This study examined the effect of two freeze-drying control strategies, stepwise and rapid, which differed in how pressure was reduced and shelf temperature increased. Final shelf temperatures of 20, 30 and 40 °C were tested. Retention and HPLC profiles of phenolic compounds and carotenoids were assessed, and these changes were related to colour and powder performance (moisture content, density/flow indices and water interaction characteristics).

## 2. Materials and Methods

### 2.1. Chemicals and Reagents

Standards of hydroxybenzoic acids (gallic, protocatechuic, 4-hydroxybenzoic, vanillic, syringic and ellagic acids), hydroxycinnamic acids (caffeic, trans-p-coumaric, ferulic, sinapic, trans-cinnamic and trans-2-hydroxycinnamic acids), caffeoylquinic acids (chlorogenic and neochlorogenic acids), flavan-3-ols ((+)-catechin, (−)-epicatechin and epigallocatechin), flavonols (quercetin, kaempferol, rutin, resveratrol and ethyl protocatechuate), carotenoids (fucoxanthin, violaxanthin, neoxanthin, astaxanthin, lutein, zeaxanthin, canthaxanthin, β-apo-8-carotenal, β-cryptoxanthin, α-carotene, β-carotene and lycopene), Trolox and 2,2-diphenyl-1-picrylhydrazyl (DPPH) with purity > 98% together with methanol (HPLC gradient grade), hexane (≥95%) and acetonitrile (gradient grade) were purchased from Merck (Darmstadt, Germany).

### 2.2. Sample Preparation

Pumpkin fruit (*Cucurbita maxima* Duchesne) was purchased in a local market (Pardubice, Czechia). The species identification was based on the information provided by the seller. The seedless pumpkin flesh without peel was cut into approximately uniform slices (≈3.0 cm × 4.5 cm) with a thickness of 7–10 mm. The slices were stored at −25 °C prior to freeze-drying.

### 2.3. Freeze-Drying of Pumpkin Flesh

Freeze-drying was performed in a laboratory freeze-dryer (L4-110 Pro, Gregor Instruments, Sázava, Czech Republic) equipped with a condenser operating at a constant temperature of −110 °C. Samples were placed on stainless-steel trays and the trays were positioned on three shelves (shelf diameter 25 cm). The upper (fourth) shelf was intentionally left empty to standardise the drying environment across runs, as the shelf located above the product layer is known to influence heat transfer and drying dynamics [27]. All samples were arranged in a single layer, and individual pieces did not touch each other. Under the stepwise strategy (SWS), chamber pressure was reduced stepwise from 5 to 0.2 hPa, while the shelf temperature was increased from −25 °C to the final temperature of 20, 30 or 40 °C (SWS20, SWS30 and SWS40, respectively). The corresponding time profiles of chamber pressure and shelf temperature are shown in Figure S1. Under the rapid strategy (RS), the chamber pressure was reduced to ~0.2 hPa after 55 min, and the shelf temperature was raised from −25 °C directly to the final setpoint (20–40 °C; RS20–RS40). Chamber pressure was controlled via a regulating valve. Freeze-drying was conducted as a single continuous programme for each treatment. The total process time was fixed and identical for all treatments, while the pressure and shelf-temperature profiles differed between treatments. At the end of each run, the chamber was returned to atmospheric pressure by backfilling with air. The freeze-dried material was milled at 8000 rpm for 10 s and subsequently sieved through a ~500 μm mesh. The resulting powder was stored at −25 °C in vacuum-sealed plastic bags until further analysis.

### 2.4. Physical Properties and Colour

#### 2.4.1. Moisture Content Determination

Moisture content was determined using a halogen moisture analyser (KERN DLB 160-3A, KERN & SOHN GmbH, Balingen, Germany). Approximately 0.8 g of sample was dried at 105 °C until the change in moisture content was below 0.1% per minute. Moisture content was expressed in a percentage.

#### 2.4.2. Colour Determination

Colour was measured using a UltraScan VIS (Hunter Associates Laboratory Inc., Reston, VA, USA) spectrophotometer with d/8° geometry and D65 illuminant. Measurements were performed in reflectance mode with specular component excluded and reported in the CIELAB colour space as lightness (L*, 0 = black, 100 = white), a* (redness (+)-greenness (-)) and b* (yellowness (+)-blueness (-)). Chroma (C*) and hue angle (*h°*) were calculated from a* and b* using equations.

(1) $$C_{ab}^{*}=\sqrt{{\left(a^{*}\right)}^{2}+{\left(b^{*}\right)}^{2}},$$

(2) $$h^{{}^{\circ}}={tan}^{-1}\left(\frac{b^{*}}{a^{*}}\right),$$

Each sample was analysed in four replicates. Data acquisition and processing were performed in EasyMatch QC (v4.96, Hunter Associates Laboratory Inc., Reston, VA, USA). Pairwise colour differences (ΔE*_ab_) were calculated using Equation (3).

(3) $$∆E_{ab}^{*}=\sqrt{{\left(∆L^{*}\right)}^{2}+{\left({∆a}^{*}\right)}^{2}+{\left({∆b}^{*}\right)}^{2}}$$

#### 2.4.3. Powder Flowability

Bulk density (*ρ*_bulk_, g/cm^3^) was determined by gently filling a 10 mL graduated cylinder with powder and calculating the mass-to-volume ratio. For tapped density (*ρ*_tapped_, g/cm^3^), the same cylinder was then tapped 125 times, after which the density was calculated from the new settled volume [28]. Both Carr index (Equation (4)) and Hausner ratio (Equation (5)) were calculated from bulk and tapped densities as:

(4) $$CI=\frac{\rho_{tapped}-{\;\rho }_{bulk}}{\rho_{tapped}},$$

(5) $$HR=\frac{\rho_{tapped}}{\rho_{bulk}}.$$

#### 2.4.4. Water Interaction Properties

Water-holding capacity (WHC), swelling capacity (SWC) and water solubility index (WSI) were determined with minor modifications to the method described by Izli et al. [28]. For WHC, approximately 0.6 g of freeze-dried pumpkin powder (*m*_1_) was weighed into a centrifugation tube (*m*_0_). Distilled water (10 mL) was added, and the mixture was thoroughly homogenised by shaking. The tube was then placed in a water bath (60 °C, 30 min). After cooling to ambient temperature, the sample was centrifuged at 4830× *g* for 20 min (Sorvall ST4R Pro-MD, Thermo Fisher Scientific Inc., Waltham, MA, USA). The supernatant was carefully decanted, and the tube containing sediment was weighed (*m*_2_). Each sample was analysed in four replicates. WHC was calculated using Equation (6) and was expressed on a dry weight basis (g/g DW):

(6) $$WHC=\frac{m_{2}-m_{0}}{m_{1}}.$$

Swelling capacity (SWC) was determined as follows: approximately 0.8 g of freeze-dried sample (*m*) was added to a graduated cylinder (*V*_1_) and wetted by 10 mL of distilled water. The mixture was carefully mixed with a spatula and left in a dark cabinet for 24 h after which the volume was noted (*V*_2_). SWC was calculated using Equation (7).

(7) $$SWC=\frac{V_{2}-V_{1}}{m}.$$

Approximately 0.2 g of freeze-dried powder was mixed with 12 mL of distilled water in a 15 mL centrifuge tube. The tube was heated at 80 °C for 30 min in a water bath, cooled and the supernatant (after centrifugation at 4830× *g*, 20 min) was carefully placed into a pre-weighed (*m*_0_*)* evaporating dish and dried at 105 °C for 24 h. The dish containing the dried residue was weighed (*m*_1_). Solubility index was calculated according to Equation (8).

(8) $$WSI=\frac{m_{1}-m_{0}}{m_{1}}\times 100.$$

### 2.5. Phytochemical Extraction and HPLC Analysis

#### 2.5.1. Extraction of Phenolic Compounds

Individual phenolic compounds were extracted according to the method described by Shao et al. [29] with minor modifications. Briefly, 0.2 g of finely ground pumpkin powder was mixed with 10 mL of a methanol/water solution (90:10, *v*/*v*) and subjected to ultrasonic-assisted extraction at 40 °C for 15 min. The resulting mixture was then centrifuged (Hettich EBA 20, Andreas Hettich, Tuttlingen, Germany) at 4321× *g* for 20 min. Subsequently, the supernatant was collected and filtered through a 0.22 µm nylon syringe filter prior to analysis. Each sample was extracted in duplicate.

#### 2.5.2. HPLC Analysis of Phenolic Compounds

The profile of individual phenolic compounds was determined using an HPLC system (Thermo Scientific Dionex Ultimate 3000, Thermo Fisher Scientific Inc., Waltham, MA, USA) equipped with a diode array detector (DAD-3000RS), a rapid separation autosampler, a binary pump (HPG-3x00RS), and a solvent selector valve (HPG-3400RS). Data acquisition and processing were performed using Chromeleon™ 7.2 Chromatography Data System (Thermo Scientific Scientific Inc., Waltham, MA, USA). Phenolic compounds were analysed according to Deng et al. [30] with minor modifications. Separation was achieved on a Kinetex C18 column (150 × 4.6 mm, 2.6 µm; Phenomenex, distributed by Chromservis, Prague, Czech Republic). A sample volume of 10 µL was injected and eluted under gradient conditions using (A) redistilled water/acetic acid (99:1, *v*/*v*) and (B) redistilled water/acetonitrile/acetic acid (67:32:1, *v*/*v*/*v*). The gradient programme was as follows: 0 min, 10% B; 0–10 min, 10–20% B; 10–16 min, 20–40% B; 16–20 min, 40–50% B; 25–26 min, 50–70% B; 26–30 min, 70% B; 30–40 min, 70–10% B; and 40–45 min, 10% B. The flow rate was set at 1.0 mL/min, and the column temperature was maintained at 30 °C. Detection was carried out at 275 nm. The DAD response was linear for all analysed phenolic acids within the calibration range of 0.05–120.0 mg/L, with correlation coefficients (R^2^) exceeding 0.9988. Individual phenolic compounds were tentatively identified based on their retention times and confirmed by the method of standard addition. Each extract was analysed in duplicate (overall *N* = 4).

#### 2.5.3. Antioxidant Capacity of Pumpkin Powder

Antioxidant capacity was determined using stable radial 2,2-diphenyl-1-picrylhydrazyl (DPPH) assay adopting the procedure used in our previous study [31]. Briefly, 5.0 mL of DPPH methanolic solution (12.5 mg in 500 mL) was mixed with 300 µL of sample extract (see chapter 2.5.1) and incubated for 10 min in a dark cabinet. Then, a decrease in absorbance at 517 nm was monitored in UV-2600 (Shimadzu, Kyoto, Japan). The results were expressed as Trolox equivalent (TE) on a dry weight basis (µg/g DW).

#### 2.5.4. Extraction of Carotenoids

Carotenoids were extracted under reduced light conditions to prevent degradation. Briefly, 0.01 g of freeze-dried pumpkin powder was weighed into dark glass vials, and 4 mL of 0.1 M NaCl solution was added. The mixture was agitated on a shaker for 30 min at room temperature. Subsequently, 3 mL of hexane containing 0.05% BHT and 2 mL of isopropanol were added, and the samples were further shaken for 15 min at room temperature. The resulting extract was transferred using a Pasteur pipette into Eppendorf tubes and centrifuged at 21,300× *g* for 10 min (Velocity 13µ, Dynamica Scientific Ltd., Livingston, UK). The upper organic phase was collected, while the remaining pellet was re-extracted using 3 mL of hexane with 0.05% BHT and 2 mL of isopropanol following the same procedure. The extraction step was repeated, and the collected supernatants were pooled. The combined extracts were rapidly evaporated to dryness in a water bath, and the residue was reconstituted in isopropanol and quantitatively transferred into a 10 mL volumetric flask. Afterwards, samples were filtered through nylon filters (13 mm, 0.45 µm) and injected into the HPLC system. The extraction procedure was performed in duplicate.

#### 2.5.5. HPLC Analysis of Carotenoids

Carotenoid content was determined using an HPLC system (Thermo Scientific Dionex Ultimate 3000, Thermo Fisher Scientific Inc., Waltham, MA, USA) equipped with a diode array detector (DAD-3000RS). The analysis was performed according to the method described by Gebregziabher et al. [32] with minor modifications. Separation was achieved on a YMC Carotenoid S-5 column (250 × 4.6 mm, 5 µm; YMC Co., Ltd., Komatsu, Japan). The injection volume was 50 µL, the flow rate was set at 0.7 mL/min, and the column temperature was maintained at 25 °C. The mobile phase consisted of (A) methanol and (B) methanol/methyl tert-butyl ether (MTBE) (20:80, *v*/*v*). The gradient elution programme was as follows: 0–10 min, 90–75% A; 10–20 min, 75–50% A; 20–25 min, 50–30% A; 25–35 min, 30–10% A; 35–37 min, 10–6% A; 37–39 min, 6–90% A; and 39–50 min, 90% A. The total run time was 50 min. Detection was carried out at 420 nm. The DAD response was linear within the calibration range of 0.05–10.0 µg/mL, with correlation coefficients (R^2^) exceeding 0.9975. Individual carotenoids were identified based on their retention times and further confirmed using the method of standard addition. Each extract was analysed twice, resulting in overall *N* = 4.

### 2.6. Scanning Electron Microscopy

The samples were sputter-coated with a 2 nm gold layer using a Q150R Plus rotary pumped coater (Quorum Technologies Ltd., Laughton, UK) to render the surface electrically conductive. SEM pictures were acquired in backscattered electron mode (BSE) at an accelerating voltage of 10 kV and a magnification of 300× using VEGA3-SBU equipment (Tescan Orsay Holding, Brno, Czech Republic).

### 2.7. Statistical Analysis

Two-way analysis of variance (ANOVA) was used to evaluate the effects of drying regime (Factor A: stepwise vs. rapid) and final shelf temperature (Factor B: 20, 30 and 40 °C), including their interaction, on each measured response variable. Tukey’s post hoc pairwise comparisons were performed for each variable to identify differences between individual treatment levels using R (emmeans package, *α* = 0.05). The multivariate structure of the dataset was explored using principal component analysis (PCA) computed by singular value decomposition (SVD). This numerically stable approach was applied to the centred and scaled data matrix and provides orthogonal linear combinations of the original variables [33]. SVD is applicable to general (including non-square) data matrices and is widely used for dimensionality reduction, which suits the present multivariate dataset [34]. Analyses were performed in R (FactoMineR and factoextra). The number of retained dimensions was guided by a scree plot (explained variance vs. dimension), variable representation on the selected dimensions was assessed using cos^2^, and results were visualised using a biplot of the first two dimensions with 95% confidence ellipses for sample groupings.

## 3. Results and Discussion

### 3.1. Moisture Content and Colour of Freeze-Dried Pumpkin Powder

In our study, systematically higher moisture contents were observed for SWS (6.2–8.8%) compared with RS (4.7–5.4%) samples across all shelf temperatures, suggesting that the rapid procedure promoted more complete dehydration under the applied fixed-time protocol (Table 1). Jakubczyk and Nowak [11] showed that a stepwise shelf-temperature programme can extend freeze-drying time compared with a more intensive constant-temperature approach, suggesting that gentler profiles do not necessarily achieve the driest endpoint.

Bulk density is a practical packing indicator; higher bulk density typically reduces the volume required for storage and transport and often reflects less aerated packing. Bulk density was higher for SWS than for RS samples (*p* < 0.001) and reached its maximum in SWS20 (0.178 g/cm^3^). Our bulk density values were comparable to those reported for freeze-dried pumpkin waste [35], but lower than values reported for freeze-dried pumpkin flesh and skin [9] and pumpkin chips [5]. The lower density in the RS powders may be related to their higher residual moisture, which, as suggested by Lim et al. [9], can be associated with the formation of a more open particle structure. Köprüalan et al. [5] reported that decreasing chamber pressure during freeze-drying led to lower bulk density. This may also help explain our results, as the lowest pressure (≈0.2 hPa) was maintained for a shorter period in the SWS runs than in the RS runs. Tapped density reflects how efficiently a powder consolidates under handling, which is relevant for packaging and transport. High *ρ_tapped_* value means that more product mass fits into the given container volume. Tapped density was consistently higher for SWS than for RS (*p* < 0.001), and within the stepwise schedule it decreased from 0.265 to 0.193 g/cm^3^ as the final shelf temperature increased (*p* < 0.001), with the lowest values observed in SWS40 and RS40. All powders showed poor flowability (CI 0.22–0.338; HR 1.29–1.51), but a clear improvement with increasing final shelf temperature was observed for SWS (CI from 0.338 to 0.22; HR from 1.51 to 1.29 with the increase in temperature from 20 to 40 °C), while RS improved mainly at 40 °C (CI from 0.33 to 0.27; HI from 1.49 to 1.38).

Our findings are consistent with those of Stajčič et al. [35], who reported similar densities, Carr index and Hausner ratio values and classified the resulting freeze-dried pumpkin pulp powder as having very poor flowability. This is also supported by the SEM micrographs (Figure S2), which show mainly irregular, plate-like particles with sharp edges and occasional agglomeration. Such flake-shaped fragments can catch on one another and increase interparticle friction and cohesion, consistent with the high CI and HR values and the poor flowability of the powders. No systematic differences in particle morphology were apparent across drying strategies (SWS vs. RS) or final shelf temperatures in the SEM images.

Beyond flowability, reconstitution performance is equally critical for powdered ingredients, as it determines how readily the powder disperses and hydrates in water. Water-holding capacity (WHC) increased with final shelf temperature under both strategies, rising from 9.5 to 10.9 g/g DW for SWS (*p* < 0.01) and from 9.3 to 10.2 g/g DW for RS (*p* < 0.01). In contrast, swelling capacity (SWC) decreased as final shelf temperature increased, dropping from 9.1 to 7.9 mL/g DW for SWS (*p* < 0.001) and from 6.9 to 5.84 mL/g DW for RS (*p* < 0.001). Moreover, SWC was consistently higher for SWS than for RS (*p* < 0.05). In contrast, WSI remained within a narrow range of about 42–46%. The drying conditions did not show a clear effect on this parameter. A similar observation was reported for carrier-free fruit and vegetable powders, where the composition of the raw material was more important for reconstitution behaviour than the drying method [36]. The limited changes in WSI may be related to the native structure of pumpkin cell-wall polysaccharides and dietary fibre. Freeze-drying is a low-temperature process and likely did not cause sufficient depolymerisation of pectin, cellulose, and hemicellulose to increase the amount of water-soluble material. Que et al. [37] also reported markedly lower water solubility for freeze-dried pumpkin flour than for hot-air-dried flour, suggesting that thermal treatment, rather than freeze-drying itself, promoted the solubilisation of pumpkin components. Similarly, Marçal et al. [38] found that drying did not significantly change the fibre content of mango peel, while only small changes were observed in the carbohydrate composition of its soluble and insoluble fibre fractions. Thus, the narrow WSI range observed in the present study suggests that freeze-drying and the applied pressure treatment did not substantially disrupt the fibre-rich pumpkin matrix or release additional soluble polysaccharide fractions. In a previous study, both water-holding capacity and swelling properties of pumpkin powder decreased as the drying temperature increased from 60 to 80 °C [28]. In our study, WHC and swelling capacity tended to vary in opposite directions, although no significant correlation was detected. WHC and swelling capacity reflect different hydration mechanisms and may therefore respond independently [39].

Colour differences were evident between drying strategies and final shelf temperatures. Lightness (L*) decreased with increasing final shelf temperature under both strategies, but the decline was more pronounced for the stepwise schedule, i.e., from 65.8 to 62.03 (*p* < 0.001), indicating noticeably darker powders at 40 °C. On the other hand, rapid-dried samples remained comparatively lighter (67.2 for RS20 and 65.4 for RS40). Redness (a*) was higher for SWS (≈8.5–9.0) than for RS (≈6.6–7.6), while yellowness (b*) showed only minor variation across treatments (≈22.0–23.4). Chroma (C*_ab_) decreased slightly at higher shelf temperatures, particularly for SWS40, suggesting a modest loss of colour saturation. In contrast, the hue angle (*h°*) was higher for RS (≈71.1–73.2) than for SWS (≈69.0–70.2), implying a small shift towards a more yellow-toned appearance in the rapid-dried powders. Although several individual CIELAB coordinates differed significantly between treatments, the practical relevance of these differences was evaluated using the overall colour difference ΔE*_ab_ (Figure S3). The ΔE*_ab_ values ranged from 1.28 to 5.28. Fernández-Vázquez et al. [40] reported that colour differences become detectable at around ΔE*_ab_ ≈ 1.5 for trained assessors, whereas untrained observers typically require a larger difference (around ΔE*_ab_ ≈ 2.8). In this context, many of the contrasts in our dataset would be expected to be only weakly perceptible, particularly for untrained observers. By contrast, the largest differences (approaching ΔE*_ab_ ≈ 5) are likely to be clearly visible to both trained and untrained panels.

### 3.2. The Effect of Freeze-Drying Strategy on Phenolic Content

**Figure 1:**
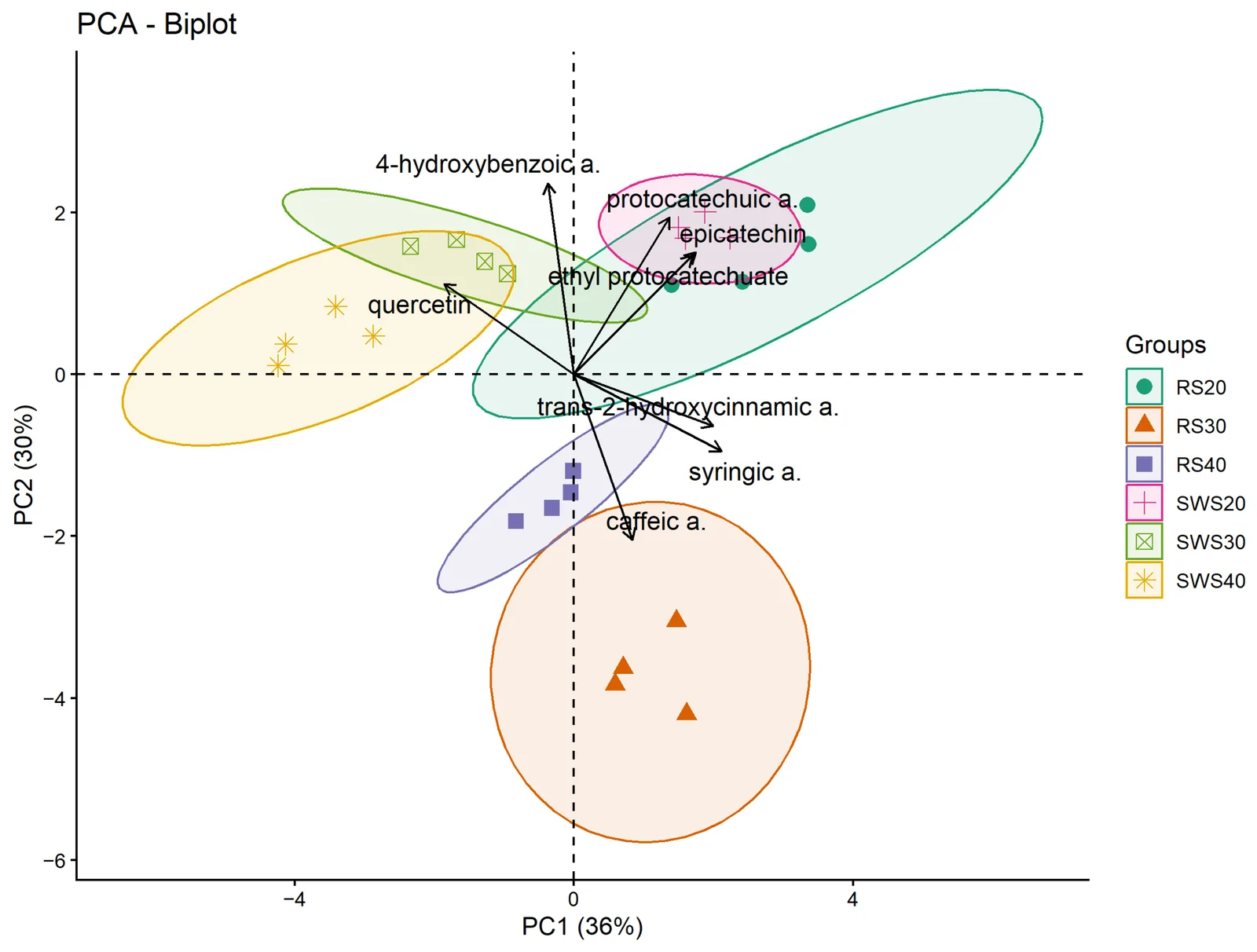
Biplot of phenolic compounds in freeze-dried pumpkin powder. Points represent individual samples freeze-dried using the rapid strategy (RS) or stepwise strategy (SWS) at final shelf temperatures of 20, 30 and 40 °C. Variables with cos^2^ ≥ 0.65 are displayed. Ellipses denote 95% confidence regions for the group dispersion in the PC1–PC2 space.

In total, 14 phenolic compounds were identified and quantified in the freeze-dried pumpkin powders (Table 2). Among the quantified phenolic compounds, protocatechuic acid was the major constituent (88.3–130.3 µg/g DW), exceeding the levels of vanillic acid, (–)-epicatechin and sinapic acid, which were detected at around 30 µg/g DW. Protocatechuic acid accounted for ~43% of the sum of identified phenolics across all the freeze-dried conditions. Regarding the effect of final shelf temperature and drying strategy, protocatechuic acid was significantly influenced by both shelf temperature (*p* < 0.001) and freeze-drying strategy (*p* < 0.001). Protocatechuic acid reached its highest concentration in the sample produced using the stepwise protocol with a final shelf temperature of 20 °C (130 ± 2 μg/g DW), and its level decreased progressively as the final shelf temperature increased, resulting in an approximately 26% reduction in SWS40 (*p* < 0.001). The same pattern was observed for the rapid protocol. However, concentrations were consistently lower than those obtained under stepwise conditions (*p* < 0.01). (-)-Epicatechin also declined with increasing final shelf temperature (*p* < 0.001), while the drying protocol itself did not exert a detectable effect on its concentration (*p* > 0.05). In contrast, vanillic acid was influenced primarily by the drying regime. Under the stepwise schedule, the content was significantly lower for SWS30 (22.8 µg/g DW; *p* < 0.01) and SWS40 (23.0 µg/g DW; *p* < 0.05) compared with SWS20 (25.4 µg/g DW), while under the rapid schedule the vanillic acid content slightly increased with increasing final shelf temperature (*p* < 0.01). Further compounds showed divergent responses. Syringic acid, sinapic acid and trans-2-hydroxycinnamic acid levels were similar across final shelf-temperature variants under the rapid regime, while a decreasing trend with increasing shelf temperature was observed under SWS. Conversely, 4-hydroxybenzoic acid remained relatively stable at SWS conditions (5.6–5.7 µg/g DW; *p* > 0.05), whereas under the RS it was lower at RS30 and RS40 (2.6–3.48 µg/g DW; *p* < 0.001) than at RS20 (5.3 µg/g DW). Taken together, these contrasting patterns suggest that individual phenolic compounds respond differently to the applied drying conditions. Therefore, principal component analysis (PCA) was used to provide an integrated view of the multivariate shifts in the phenolic profile across treatments. A scree plot based on the singular value decomposition indicated that the first two dimensions captured 66% of the total variances in the phenolic content (Figure S4). This implies that the dominant variation among samples can be adequately described by a two-dimensional representation. Figure S5 presents the cos2, which indicates how each variable is captured by the selected dimensions. It corresponds to the squared correlation between the original configuration and its projection onto the factor map. Values closer to 1 denote a more reliable representation. The PCA biplot (Figure 1) reveals a clear separation of samples according to both final shelf temperature and drying regime. The SWS20 and RS20 powders formed a distinct cluster, positioned in the direction of higher loadings for protocatechuic acid, protocatechuic ester, and (−)-epicatechin, indicating that these compounds contributed strongly to the grouping of the low-temperature treatments. This pattern was also reflected in the DPPH results, where the 20 °C treatments showed higher radical-scavenging activity than the corresponding 40 °C samples (Table 2). The highest DPPH value was found for SWS20 (1190 ± 9 µg TE/g DW), followed by RS20 (1100 ± 15 µg TE/g DW), whereas the lowest values were observed for SWS40 and RS40 (990 ± 34 and 987 ± 8 µg TE/g DW, respectively). This finding is consistent with the general temperature sensitivity of catechol-type phenolics [14,15]. Although DPPH represents an overall antioxidant response rather than the activity of individual compounds, its decrease at higher final shelf temperatures supports the view that the low-temperature treatments better preserved antioxidant-active constituents. In contrast, SWS30 and SWS40 clustered in the upper-left quadrant, with the separation mainly associated with quercetin and 4-hydroxybenzoic acid. A different pattern was observed for RS30 and RS40, which formed clusters in the lower half of the biplot and aligned with higher contributions from caffeic acid, syringic acid, and trans-2-hydroxycinnamic acid. This may reflect higher extractability under the more intensive conditions. Rapid shifts in temperature or pressure can accelerate matrix disruption and weaken interactions between phenolics and cell-wall components, increasing the proportion of phenolic acids recovered in the extractable (“free”) fraction [38]. This interpretation is consistent with evidence that drying can shift phenolics from bound to more readily extractable forms and alter the apparent abundance of individual acids such as caffeic acid and related compounds [41].

The biplot separation between SWS30/SWS40 and RS30/RS40 likely reflects differences in thermal exposure and pressure history, not just the final shelf temperature setpoint [5,18]. In the stepwise schedule, the residence time at the final shelf temperature was comparatively short (≈720 min with gradual pressure reduction), whereas in the rapid schedule the samples remained at the target temperature for substantially longer (≈1175 min) under deep vacuum, i.e., 0.2 hPa. Differences in the time–temperature conditions may have influenced both the stability and the extractability of phenolics. This could explain why SWS30/SWS40 cluster closer to quercetin, while RS30/RS40 align more strongly with the hydroxy acids. In support of a time–temperature stability component, Ferreyra et al. [15] monitored individual phenolics in grape cane extracts every two weeks over three months and reported that exposure at 25 and 40 °C accelerated degradation kinetics of (+)-catechin and (−)-epicatechin in comparison with those stored at −20 and 5 °C. Bayram et al. [41] quantified free and insoluble-bound fractions and showed that drying altered their relative contributions, with the free fraction becoming more dominant in dried samples. This change was associated with weakened attachment of bound phenolics to cell-wall components. Altogether, these results support our interpretation that the opposing PCA directions of quercetin and hydroxy acids may be linked to different processes. One is related to compound stability and the second to process-driven release from the matrix, which affects extractability.

### 3.3. The Effect of Freeze-Drying Strategy on Carotenoid Content

**Figure 2:**
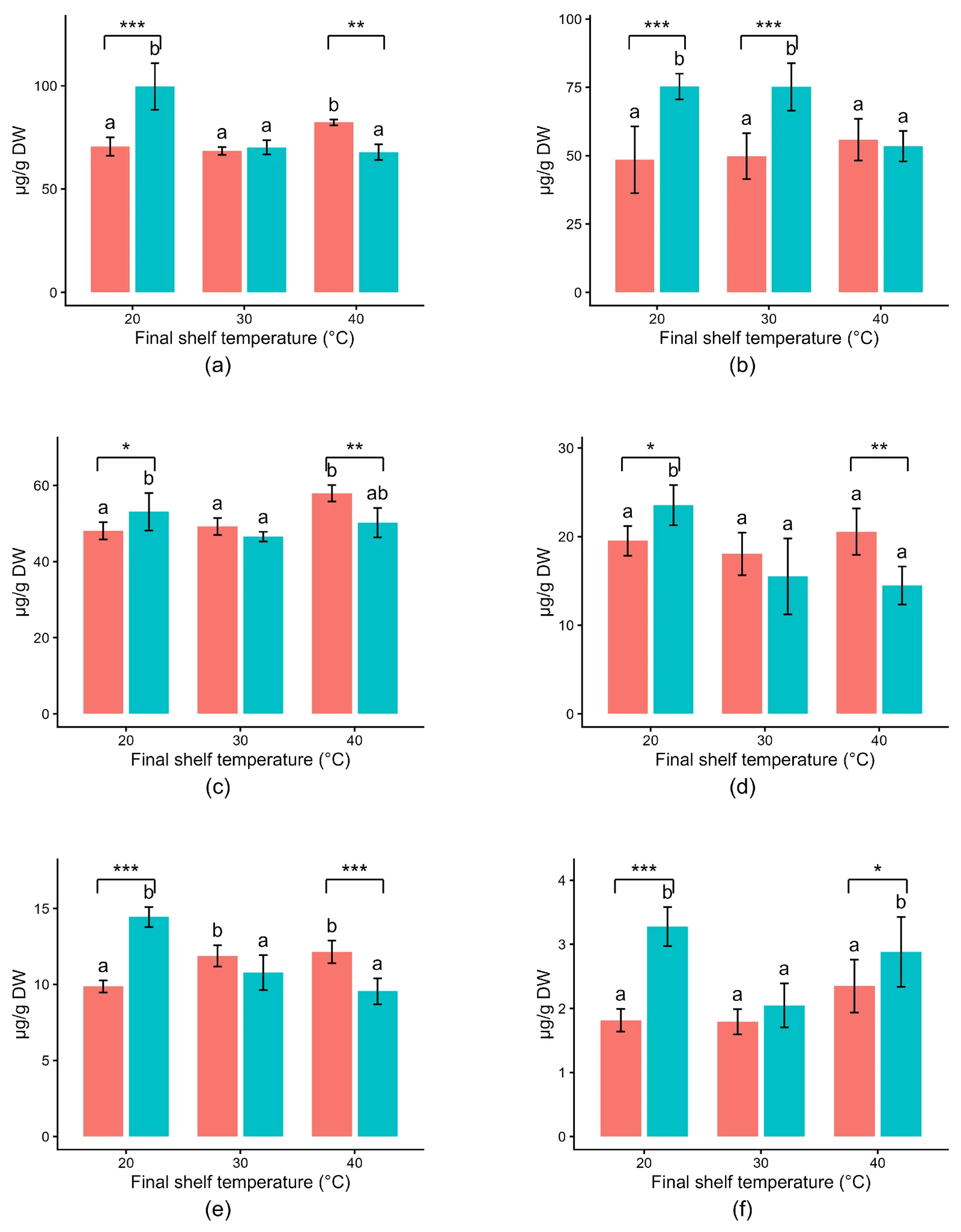
Effect of freeze-drying strategy (stepwise strategy, SWS, red columns; rapid strategy, RS, blue columns) and final shelf temperature on the content of (**a**) lutein, (**b**) β-cryptoxanthin, (**c**) β-carotene, (**d**) astaxanthin, (**e**) neoxanthin and (**f**) zeaxanthin. Values are shown as mean ± standard deviation (*N* = 4). Lowercase letters indicate significant differences among final shelf temperatures within the same drying strategy only (*p* < 0.05); therefore, letters should be compared only among columns of the same colour. Asterisks indicate significant differences between SWS and RS at the same final shelf temperature (* *p* < 0.05, ** *p* < 0.01, *** *p* < 0.001).

Across all samples, lutein was the predominant carotenoid (68–100 µg/g DW), followed by β-cryptoxanthin (49–75 µg/g DW) and β-carotene (47–58 µg/g DW). In pumpkin products and by-products, lutein and β-carotene are repeatedly reported among the major carotenoids, with xanthophylls (including lutein) often dominating in some pumpkin cultivars [1,2]. In particular, a recent study on pumpkin powder reported lutein as the most abundant saponified carotenoid, with β-carotene and β-cryptoxanthin also present among the leading components [3]. In our study, an initial SVD-based exploration indicated that the first two dimensions accounted for 57.5% and 16.5% of the total variance, respectively, in the carotenoid’s dataset, suggesting a relatively simple variance structure (Figure S6). Based on the cos^2^ values, lutein, β-cryptoxanthin, β-carotene, neoxanthin and astaxanthin were well represented on the first two PCA dimensions (cos^2^ > 0.65), whereas zeaxanthin was not (Figure S7). In contrast to the phenolic profile, the biplot did not reveal distinct clustering for carotenoids (Figure S8). Therefore, two-way ANOVA was used to test the effects of drying regime and final shelf temperature. It showed that lutein was significantly affected by final shelf temperature (*p* < 0.001) and drying strategy (*p* < 0.05). Under the SWS, lutein levels were lower at 20 and 30 °C (70.6 and 68.4 µg/g DW, respectively) and increased at 40 °C (82 µg/g DW; *p* < 0.01), while under the rapid strategy lutein was highest at 20 °C (~99 µg/g DW) and decreased at 30 and 40 °C (Figure 2a). For β-cryptoxanthin, a significant effect of drying strategy was observed (*p* < 0.001) together with a strategy × temperature interaction, whilst the main effect of final shelf temperature was not significant. This indicates that the difference between stepwise and rapid drying depended on the temperature level rather than following a uniform temperature trend (Figure 2b). Finally, β-carotene was significantly affected by final shelf temperature and by the strategy × temperature interaction (both *p* < 0.01), although the main effect of drying strategy was not significant. Β-carotene increased at 40 °C under the stepwise schedule, while no clear temperature-related differences were observed under the rapid schedule (Figure 2c). At 20 °C, the rapid strategy resulted in higher concentration of all six carotenoids than the stepwise strategy (*p* < 0.05). Thus, the effect of drying strategy was most evident at the lowest shelf temperature. This behaviour may reflect differences in oxidative exposure during the early stages of drying. Under the stepwise strategy, the chamber remained at a comparatively higher pressure for longer before reaching deep vacuum (~0.2 hPa), while under the rapid strategy the pressure was reduced to ~0.2 hPa shortly after the start of the run (after 55 min). The lower concentrations observed for SWS at 20 °C may be related to the longer exposure time at higher pressure. Oxidative degradation of all-trans carotenoids could have contributed to this effect; however, the role of residual oxygen cannot be confirmed because the oxygen concentration in the chamber was not measured. Consistent with this interpretation, Atencio et al. [4] showed that oxygen availability strongly affected the degradation of all-trans carotenoids in a pumpkin-concentrate beverage during storage for 36–42 days at different temperatures, and they further reported that oxygen depletion was least pronounced at lower temperatures (10 and 20 °C).This temperature dependence may also help explain why the strategy-related differences were most evident at 20 °C in our experiment, while the pattern at 30 and 40 °C was less clear. At a final shelf temperature of 30 °C, β-cryptoxanthin was the only carotenoid showing a clear effect of drying strategy. Its concentration was significantly higher after rapid drying than after the stepwise drying (*p* < 0.001) (Figure 2b). At 40 °C, lutein, β-carotene, astaxanthin, and neoxanthin were present at higher concentrations after stepwise drying than after rapid drying (*p* < 0.01, Figure 2a,c–e), suggesting a compound-specific temperature response that depended on the drying strategy. This may be explained by the longer exposure to 40 °C in the rapid strategy. In this treatment, the samples remained at the final shelf temperature for about 7.6 h longer, which may have promoted thermal and oxidative changes. From a kinetic perspective, prior work in model carotenoid systems indicates that moderate temperature increases can markedly accelerate degradation, with zeaxanthin showing a stronger temperature sensitivity than lutein (e.g., a decrease in half-life from 11.13 to 3.51 h for zeaxanthin at 25 and 35 °C, compared with 4.15 to 2.01 h for lutein at the same temperatures) [13]. Such kinetics provide a useful frame for interpreting the distinct temperature responses observed in our experiment. It should be noted that concentrations measured in powders also reflect matrix-specific factors such as oxygen availability, isomerisation, and extractability. In this context, the longer residence at 40 °C during the rapid schedule could still be expected to impose a measurable thermal burden, consistent with reports for astaxanthin solutions showing non-negligible changes within hours at 40 °C [12]. Zeaxanthin did not show the same response as several other carotenoids at 40 °C. This indicates that the effect cannot be explained only by the final shelf temperature. The different drying schedules may also have affected zeaxanthin stability or its release from the matrix.

## 4. Conclusions

The results showed that both drying programme and final shelf temperature affected pumpkin powder properties and antioxidant retention. The rapid procedure gave powders with lower residual moisture than the stepwise programme. Stepwise drying led to higher bulk and tapped densities and stronger swelling. Flowability remained poor in all samples, but it improved when the final shelf temperature was higher. Colour changes were statistically detectable yet mostly of limited practical relevance (ΔE*_ab_ 1.28–5.28). Phenolic compounds did not respond to processing in the same way. In the multivariate analysis, samples dried at 20 °C were associated with higher levels of protocatechuic acid, ethyl protocatechuate and (–)-epicatechin. At higher final shelf temperatures, the samples were separated mainly according to the drying programme. Lutein, β-cryptoxanthin and β-carotene were the dominant carotenoids. The rapid strategy was more favourable at 20 °C, where all measured carotenoids were higher. At 30 and 40 °C, the response differed among individual carotenoids. From a processing point of view, these results show that the pressure and shelf-temperature profile can influence not only residual moisture, but also powder functionality and the retention of antioxidant-related compounds. This may be useful for further optimisation of freeze-drying programmes for pumpkin and similar vegetable powders. However, the interpretation has some limitations. We used a fixed drying time and did not measure microstructure, particle size distribution, product temperature or oxygen availability during drying. Therefore, it is not clear whether the observed differences were caused mainly by degradation of sensitive compounds or by changes in their release and extractability from the plant matrix. Future work should address this by separating free and matrix-bound phenolic fractions, for example, through sequential extraction of free, esterified and insoluble-bound phenolics. Such an approach would help clarify whether changes during freeze-drying mainly reflect loss of active compounds or improved recovery of phenolics from the dried matrix.

## Figures and Tables

**Table 1:** Physical properties and colour of freeze-dried pumpkin powder.

	SWS20	SWS30	SWS40	RS20	RS30	RS40
MC	6.7 ± 0.3 ^c^	6.2 ± 0.3 ^c^	8.8 ± 0.4 ^d^	5.3 ± 0.2 ^ab^	5.4 ± 0.2 ^b^	4.7 ± 0.4 ^a^
*ρ_bulk_*	0.178 ± 0.005 ^e^	0.16 ± 0.01 ^d^	0.150 ± 0.000 ^cd^	0.135 ± 0.006 ^ab^	0.143 ± 0.005 ^bc^	0.123 ± 0.005 ^a^
*ρ_tapped_*	0.265 ± 0.006 ^e^	0.22 ± 0.01 ^d^	0.193 ± 0.005 ^b^	0.203 ± 0.006 ^bc^	0.215 ± 0.006 ^cd^	0.170 ± 0.000 ^a^
CI	0.338 ± 0.006 ^d^	0.283 ± 0.008 ^bc^	0.22 ± 0.02 ^a^	0.33 ± 0.02 ^cd^	0.33 ± 0.03 ^cd^	0.27 ± 0.03 ^b^
HR	1.51 ± 0.01 ^d^	1.40 ± 0.01 ^bc^	1.29 ± 0.03 ^a^	1.49 ± 0.05 ^cd^	1.49 ± 0.07 ^cd^	1.38 ± 0.03 ^ab^
WHC	9.5 ± 0.4 ^ab^	10.1 ± 0.5 ^b^	10.9 ± 0.4 ^c^	9.3 ± 0.1 ^a^	9.8 ± 0.2 ^ab^	10.2 ± 0.3 ^bc^
SWC	9.1 ± 0.3 ^d^	8.57 ± 0.03 ^d^	7.9 ± 0.3 ^c^	6.9 ± 0.3 ^b^	6.9 ± 0.3 ^b^	5.84 ± 0.02 ^a^
WSI	44.8 ± 0.6 ^bc^	42.3 ± 0.5 ^a^	42.0 ± 1.0 ^a^	45.1 ± 0.8 ^bc^	45.9 ± 0.3 ^c^	44.1 ± 0.5 ^b^
L*	65.8 ± 0.2 ^c^	64.0 ± 0.7 ^b^	62.03 ± 0.08 ^a^	67.2 ± 0.3 ^d^	65.9 ± 0.4 ^c^	65.4 ± 0.5 ^c^
a*	9.0 ± 0.2 ^c^	8.5 ± 0.1 ^c^	8.5 ± 0.2 ^c^	7.4 ± 0.3 ^b^	7.62 ± 0.05 ^b^	6.6 ± 0.5 ^a^
b*	23.31 ± 0.06 ^b^	23.4 ± 0.3 ^b^	23.0 ± 1.0 ^ab^	22.3 ± 0.1 ^ab^	22.3 ± 0.6 ^ab^	22.0 ± 1.0 ^a^
C*_ab_	25.0 ± 0.1 ^c^	25.0 ± 0.2 ^c^	24.0 ± 0.6 ^bc^	23.5 ± 0.2 ^ab^	24.0 ± 0.6 ^ab^	23 ± 1 ^a^
*h°*	69.0 ± 0.3 ^a^	70.2 ± 0.5 ^bc^	69.7 ± 0.2 ^ab^	71.5 ± 0.6 ^d^	71.1 ± 0.4 ^cd^	73.2 ± 0.5 ^e^

SWS, stepwise strategy; RS, rapid strategy; 20, 30 and 40 denote final shelf temperature (°C); MC, moisture content (%); CI, Carr index; HR, Hausner ratio; WHC, water-holding capacity (g/g DW); SWC, swelling capacity (mL/g DW); WSI, water solubility (%); different letters indicate statistical differences according to Tukey’s post hoc pairwise comparison within each row (*p* < 0.05); mean ± standard deviation (*N* = 4).

**Table 2:** The content of phenolic compounds (μg/g DW) in freeze-dried pumpkin powder.

Compound						
	SWS20	SWS30	SWS40	RS20	RS30	RS40
gallic acid	6.0 ± 0.2 ^c^	6.9 ± 0.3 ^d^	4.3 ± 0.1 ^a^	7.9 ± 0.3 ^e^	6.6 ± 0.3 ^d^	5.2 ± 0.2 ^b^
protocatechuic acid	130 ± 2 ^e^	103.3 ± 0.9 ^c^	96 ± 5 ^b^	121 ± 4 ^d^	89 ± 2 ^a^	88.3 ± 0.7 ^a^
4-hydroxybenzoic acid	5.7 ± 0.2 ^b^	5.6 ± 0.1 ^b^	5.7 ± 0.1 ^b^	5.3 ± 0.9 ^b^	2.6 ± 0.4 ^a^	3.48 ± 0.07 ^a^
(+)-catechin	6.9 ± 0.3 ^b^	4.7 ± 0.3 ^a^	5.0 ± 0.5 ^a^	7.2 ± 0.8 ^b^	5.4 ± 0.9 ^a^	5.8 ± 0.6 ^ab^
vanillic acid	25.4 ± 0.3 ^bc^	22.8 ± 0.8 ^a^	23 ± 1 ^a^	25 ± 1 ^ab^	25.4 ± 0.4 ^bc^	27.1 ± 0.7 ^c^
caffeic acid	4.4 ± 0.2 ^a^	3.6 ± 0.2 ^a^	3.6 ± 0.1 ^a^	4.0 ± 0.4 ^a^	8.7 ± 0.6 ^b^	4.2 ± 0.3 ^a^
syringic acid	13.2 ± 0.3 ^c^	11.4 ± 0.3 ^b^	9.8 ± 0.8 ^a^	13.2 ± 0.5 ^c^	13.8 ± 0.7 ^c^	13.5 ± 0.4 ^c^
(-)-epicatechin	36 ± 2 ^c^	34 ± 2 ^bc^	25 ± 2 ^a^	39 ± 4 ^c^	28 ± 1 ^ab^	26.9 ± 0.9 ^a^
sinapic acid	32 ± 1 ^d^	30 ± 1 ^cd^	24.8 ± 0.9 ^a^	28 ± 1 ^bc^	26 ± 1 ^ab^	29 ± 2 ^bc^
rutin	6.0 ± 0.7 ^c^	2 ± 2 ^a^	<LOQ	<LOQ	4.3 ± 0.2 ^b^	4.33 ± 0.09 ^b^
t-2-hydroxycinnamic	0.60 ± 0.07 ^bc^	0.33 ± 0.03 ^a^	0.28 ± 0.09 ^a^	0.60 ± 0.04 ^bc^	0.66 ± 0.2 ^c^	0.4 ± 0.2 ^ab^
ethyl protocatechuate	7.7 ± 0.2 ^b^	6.2 ± 0.3 ^a^	6.4 ± 0.3 ^a^	8.8 ± 0.4 ^c^	6.1 ± 0.3 ^a^	6.3 ± 0.3 ^a^
resveratrol	2.9 ± 0.3 ^c^	3.0 ± 0.2 ^c^	2.7 ± 0.1 ^bc^	2.4 ± 0.1 ^ab^	2.12 ± 0.08 ^a^	2.7 ± 0.1 ^bc^
quercetin	10.5 ± 0.3 ^bc^	11.2 ± 0.7 ^bc^	11.8 ± 0.7 ^c^	8 ± 1 ^a^	8.2 ± 0.6 ^a^	9.7 ± 0.2 ^ab^
DPPH (µg TE/g DW)	1190 ± 9 ^d^	1045 ± 24 ^b^	990 ± 34 ^a^	1100 ± 15 ^c^	1080 ± 11 ^bc^	987 ± 8 ^a^

SWS, stepwise strategy; RS, rapid strategy; 20, 30 and 40 denote final shelf temperature (°C); TE, Trolox; different letters indicate statistical differences according to Tukey’s post hoc pairwise comparison within each row (*p* < 0.05); mean ± standard deviation (*N* = 4).

## Data Availability

The data used in this study are available from corresponding author (L.Č.) upon reasonable request.

## Supplementary Materials

The following supporting information can be downloaded at: https://www.mdpi.com/article/10.3390/antiox15080927/s1, Figure S1: Time profiles of chamber pressure and shelf temperature during freeze-drying of pumpkin flesh under the stepwise (SWS) and rapid (RS) strategies; Figure S2: SEM image of pumpkin powder produced using rapid strategy of freeze-drying at a final shelf temperature of 20 °C; Figure S3: Heatmap of pairwise colour differences (Δ*E**_ab_) between pumpkin powder produced using rapid (RS) and stepwise (SWS) freeze-drying strategies at final shelf temperatures of 20, 30, and 40 °C. The colour gradient represents the magnitude of the total colour difference between each pair of samples. Lower *ΔE*_ab_* values are shown in blue, intermediate values in light yellow-orange tones, and higher values in orange-red to red tones. The diagonal values are zero because each sample is compared with itself; Figure S4: Scree plot from the PCA (computed via singular value decomposition) for phenolics content in freeze-dried pumpkin powder; Figure S5: Squared correlation (cos^2^) values for each phenolic compound in freeze-dried pumpkin powder samples; Figure S6: Scree plot from the PCA (computed via singular value decomposition) for carotenoid content in freeze-dried pumpkin powder; Figure S7: Squared correlation (cos^2^) values for each carotenoid compound in freeze-dried pumpkin powder samples; Figure S8: Biplot of carotenoid compounds in freeze-dried pumpkin powder. Points represent individual samples freeze-dried at rapid strategy (RS) and stepwise strategy (SWS) with final shelf temperatures of 20, 30 and 40 °C. Variables with cos^2^ ≥ 0.65 are displayed. Ellipses denote 95% confidence regions for the group dispersion in the PC1-PC2 space.

## Abbreviations

The following abbreviations are used in this manuscript:

ANOVA	Analysis of variance
BHT	Butylated hydroxytoluene
CI	Carr index
DPPH	2,2-diphenyl-1-picrylhydrazyl
DW	Dry weight
HR	Hausner ratio
LOQ	Limit of quantification
MTBE	Methyl tert-butyl ether
PCA	Principal component analysis
RS	Rapid strategy of freeze-drying
SEM	Scanning electron micrograph
SVD	Singular value decomposition
SWC	Swelling capacity
SWS	Stepwise strategy of freeze-drying
TE	Trolox equivalent
WHC	Water-holding capacity
WSI	Water solubility index
