# Supplementary material for: Shelf Temperature and Depressurisation Strategy Affect Bioactive Retention and Functionality of Freeze-Dried Pumpkin Powder
Source: Antioxidants (Basel). 2026 Jul 26;15(8):927. doi: 10.3390/antiox15080927 (PMC13510251; doi:10.3390/antiox15080927)
Supplement: Supplementary file 1 [file antioxidants-15-00927-s001.zip › antioxidants-4403291-supplementary.pdf]

Shelf Temperature and Depressurisation Strategy Affect Bioactive Retention and Functionality of Freeze-Dried Pumpkin Powder

Daniela Sumczynski [1], Libor Červenka [2,*], Helena Velichová [1], Ludmila Hrabalíková [3] and Jiří Mlček [1,*]

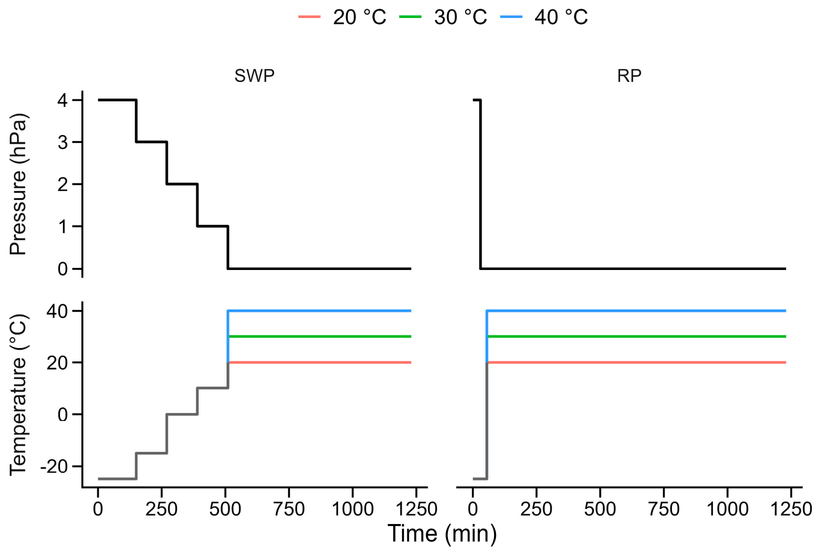

Figure S1. Time profiles of chamber pressure and shelf temperature during freeze-drying of pumpkin flesh under the stepwise (SWS) and rapid (RS) strategies.

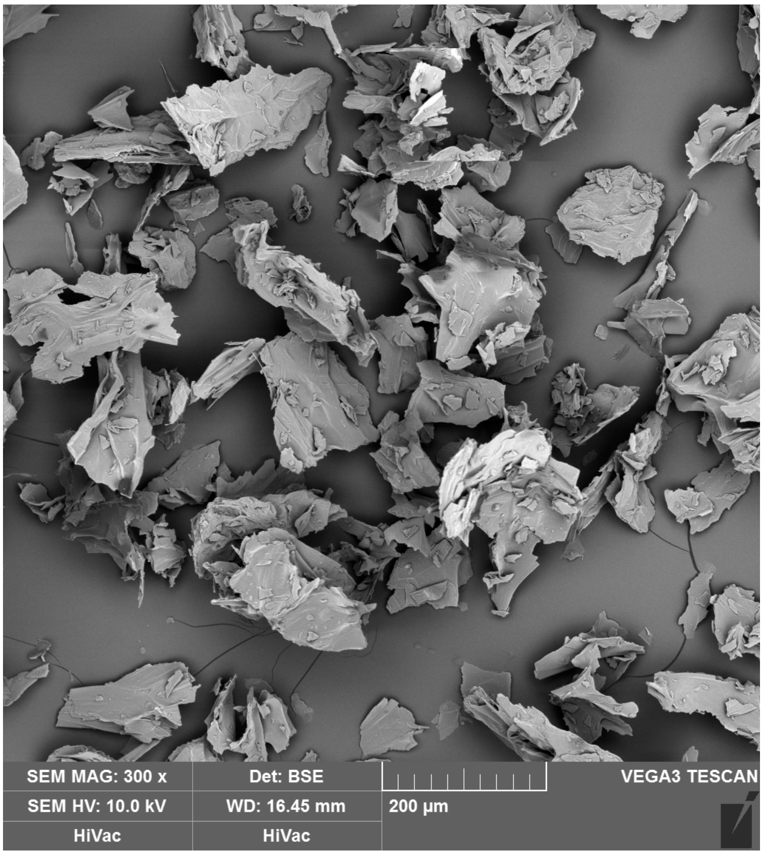

Figure S2. SEM image of pumpkin powder produced using rapid strategy of freeze-drying at a final shelf temperature of 20 °C.

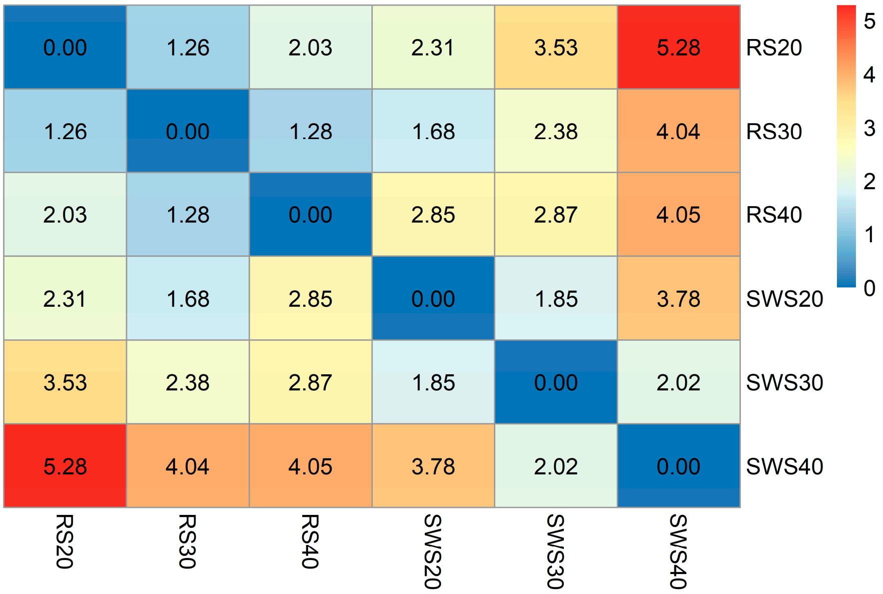

Figure S3. Heatmap of pairwise colour differences (ΔE^*_{ab}) between pumpkin powder produced using rapid (RS) and stepwise (SWS) freeze-drying strategies at final shelf temperatures of 20, 30, and 40 °C. The colour gradient represents the magnitude of the total colour difference between each pair of samples. Lower ΔE^*_{ab} values are shown in blue, intermediate values in light yellow-orange tones, and higher values in orange-red to red tones. The diagonal values are zero because each sample is compared with itself.

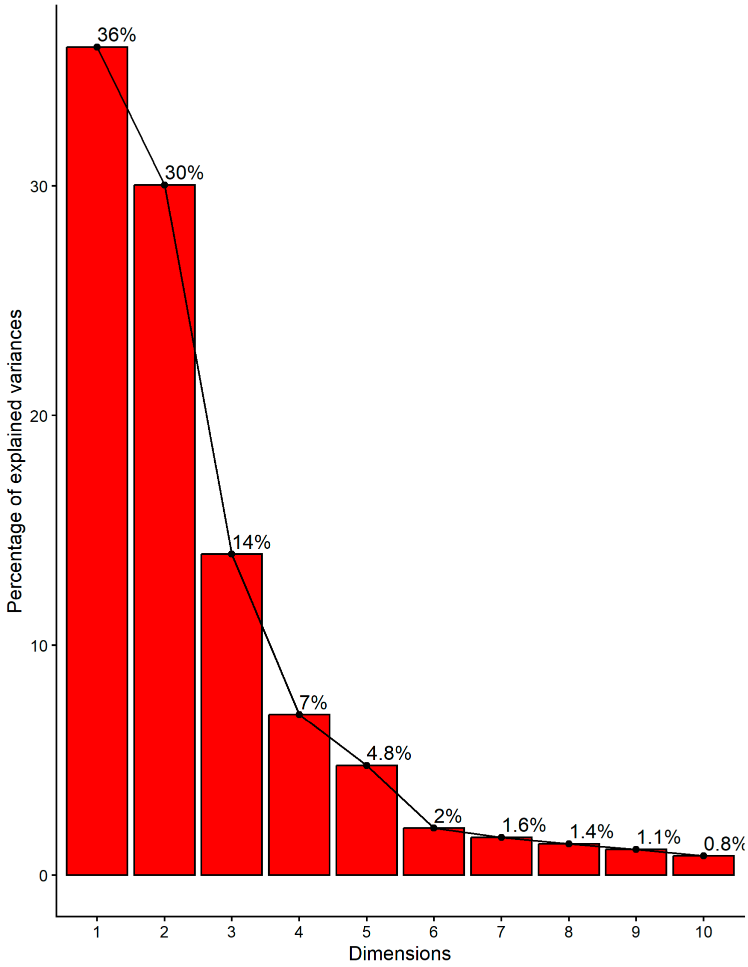

Figure S4. Scree plot from the PCA (computed via singular value decomposition) for phenolics content in freeze-dried pumpkin powder.

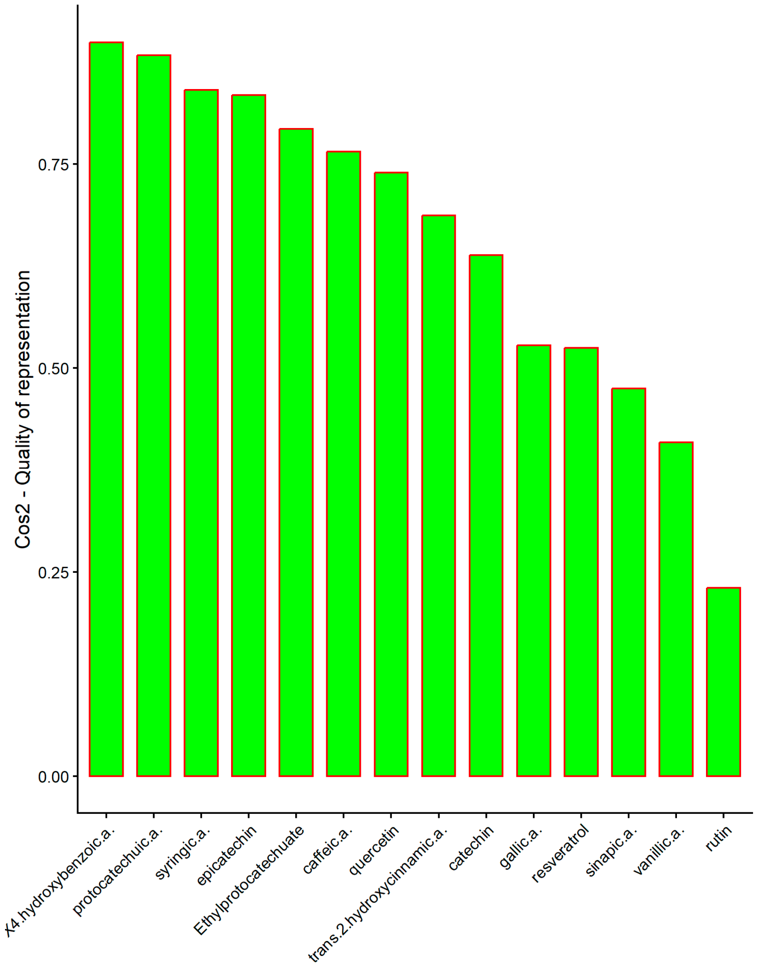

Figure S5. Squared correlation (cos^2) values for each phenolic compound in freeze-dried pumpkin powder samples.

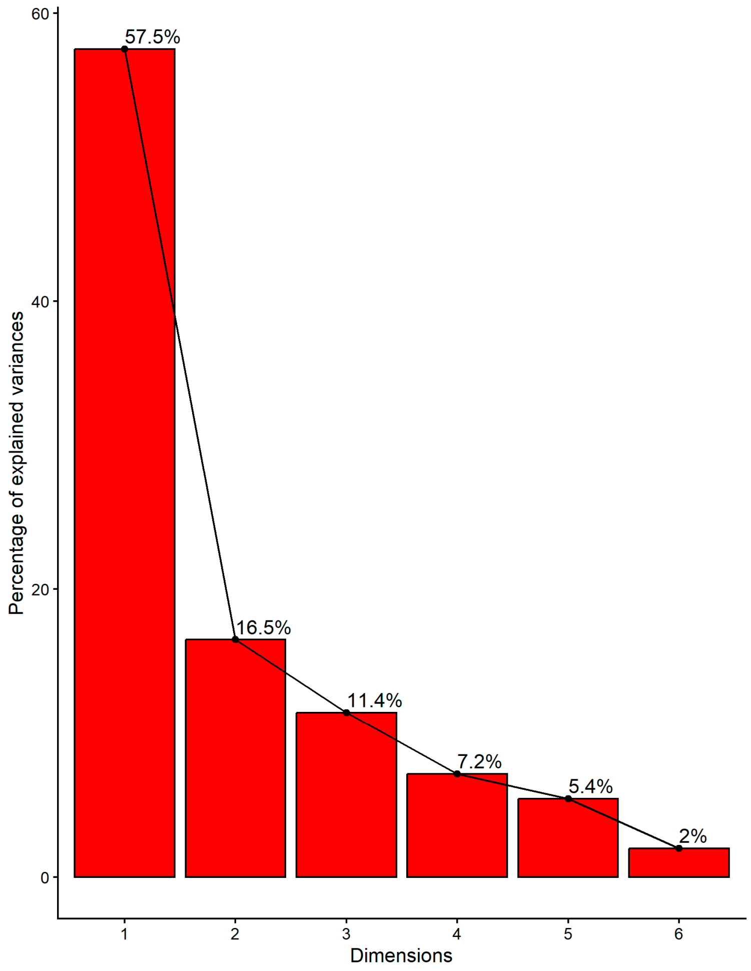

Figure S6. Scree plot from the PCA (computed via singular value decomposition) for carotenoid content in freeze-dried pumpkin powder.

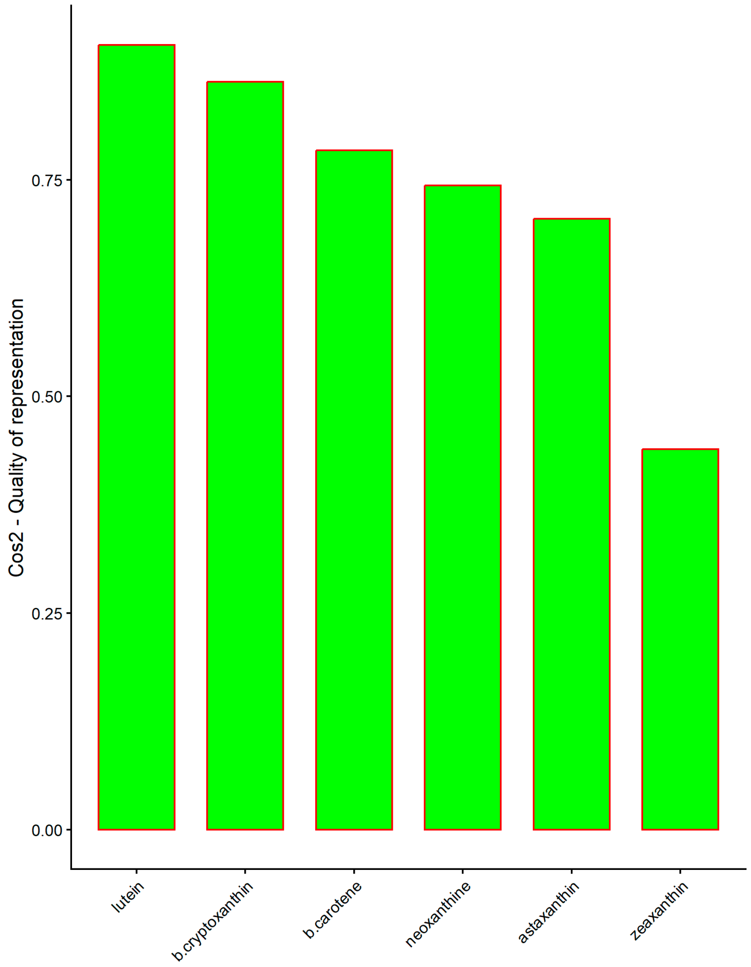

Figure S7. Squared correlation (cos^2) values for each carotenoid compound in freeze-dried pumpkin powder samples.

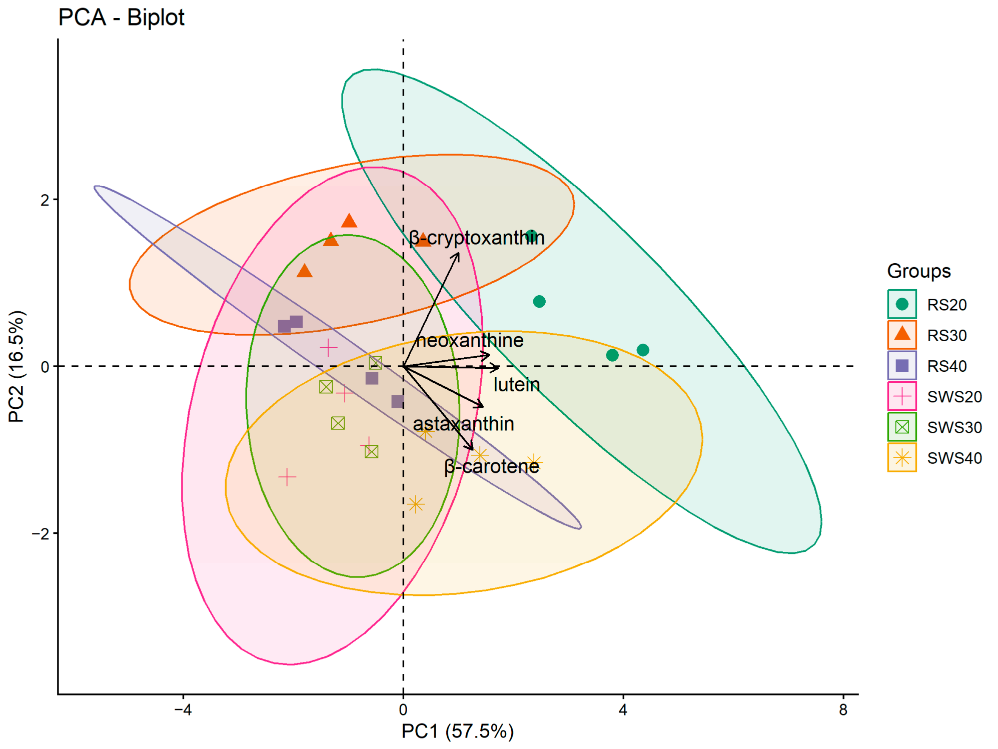

Figure S8. Biplot of carotenoid compounds in freeze-dried pumpkin powder. Points represent individual samples freeze-dried at rapid strategy (RS) and stepwise strategy (SWS) with final shelf temperatures of 20, 30 and 40 °C. Variables with $\cos^2 \geq 0.65$ are displayed. Ellipses denote 95% confidence regions for the group dispersion in the PC1-PC2 space.
